# Secondary Control: A Self-Regulatory Resource that Promotes Emotional Stability in Daily Life

**DOI:** 10.1093/geroni/igaf122.3076

**Published:** 2025-12-31

**Authors:** Matthew Pierce, Laura Klepacz, Jeremy Hamm

**Affiliations:** North Dakota State University, Fargo, North Dakota, United States; North Dakota State University, Fargo, North Dakota, United States; North Dakota State University, Fargo, North Dakota, United States

## Abstract

Although secondary control beliefs (e.g., “all clouds have a silver lining”) reflect a self-regulatory resource that protects well-being, little is known about how secondary control may help stabilize day-to-day emotional experiences and how aging might affect this regulatory process. Our study examined whether individual differences in secondary control predicted day-to-day variability in positive and negative affect, loneliness, stress, and depressed mood. We further examined how this relationship may differ across the adult lifespan. Using 1 week of daily data from a sample of adults aged 30 to 80 (n = 217), we employed multilevel heterogenous variability models to test whether higher levels of secondary control predicted less variability (the Level-1 residual variances) in emotional well-being. All models controlled for study day, age, sex, education, and financial hardship. Results showed that individuals with higher levels of secondary control experienced reduced day-to-day variability in negative affect (α=-.16, p<.005), loneliness (α=-.33, p<.005), and depressed mood (α=-.44, p<.005). Significant Age x Secondary Control interactions were observed for negative affect (α=.02, p<.005), loneliness (α=.02, p<.005), and depressed mood (α=.01, p<.005). These interactions indicated that secondary control was a stronger predictor of reduced day-to-day variability in negative affect, loneliness, and depressed mood for younger (vs. older) adults, who typically experience more volatility in their daily well-being. These findings further inform lifespan theories of development and control by documenting the self-regulatory benefits of secondary control for stabilizing day-to-day emotional well-being, especially among individuals in early adulthood and midlife.

